# Home-based educational interventions for family caregivers of older adults after stroke: a scoping review

**DOI:** 10.1590/1980-220X-REEUSP-2023-0339en

**Published:** 2024-05-17

**Authors:** Gerardo Saucedo-Pahua, Gideany Maiara Caetano, María de Jesús Jiménez-González, Jack Roberto Silva Fhon

**Affiliations:** 1 Universidad de Guanajuato, Programa de Doctorado en Ciencias en Enfermería, Celaya, Guanajuato, Mexico.; 2 Universidade de São Paulo, Programa de Pós-Graduação de Saúde do Adulto, São Paulo, SP Brazil.; 3 Universidad de Guanajuato, Celaya, Guanajuato, México.; 4 Centro Brasileiro Para o Cuidado à Saúde Baseado em Evidências: Centro de Excelência do JBI, São Paulo, SP, Brazil.

**Keywords:** Stroke, Caregivers, Aged, Education, Nonprofessional, Acidente Vascular Cerebral, Cuidadores, Idoso, Educação não Profissionalizante, Revisão, Accidente Cerebrovascular, Cuidadores, Anciano, Educación no Profesional, Revisión

## Abstract

**Objective:**

To map home-based educational interventions for family caregivers of older adults after stroke.

**Method:**

Scoping review based on the JBI methodology, carried out on May 23, 2023. The Rayyan application and Preferred Reporting Items for Systematic reviews and Meta-Analyses extension for Scoping Reviews were used.

**Results:**

Of the 1,705 studies, nine published from 2006 to 2020 were included: 44% of interventions were theoretical-practical educational; 77.7% were randomized clinical trials; and the “in-person” intervention (56%) was the most common, carried out by nurses in 88.9% of cases. Three to 15 42-minute sessions were carried out. The educational contents were organized into ten categories, divided into education aimed at caring for older adults and self-care for caregivers.

**Conclusion:**

Identified educational interventions strengthen participants’ knowledge and skills in areas such as education, care, communication, self-management, rehabilitation and nutrition as well as self-care to safely assist older adults in their activities of daily living.

## RESUMEN

**Objetivo:**

Mapear las intervenciones educativas domiciliarias para cuidadores familiares de adultos mayores después de un accidente cerebrovascular.

**Método:**

Revisión de alcance basada en la metodología del JBI realizada el 23 de mayo de 2023. Se utilizó el aplicativo Rayyan y *Preferred Reporting Items for Systematic reviews and Meta-Analyses extension for Scoping Reviews*.

**Resultados:**

De 1.705 estudios, se incluyeron nueve publicados del 2006 al 2020: el 44% de las intervenciones fueron educativas teórico-prácticas; el 77,7% fueron ensayos clínicos aleatorizados; y la intervención “cara a cara” (56%) fue el más común, entregadas por enfermeras en 88,9% de casos. Se realizaron de tres a 15 sesiones de 42 minutos. Los contenidos educativos se organizaron en diez categorías divididas en educación dirigida al cuidado del adulto mayor y al autocuidado del cuidador.

**Conclusión:**

Las intervenciones educativas identificadas fortalecen los conocimientos y habilidades de los participantes en áreas como la educación, cuidado, comunicación, autogestión, rehabilitación y nutrición, así como al autocuidado para asistir de forma segura al adulto mayor en sus actividades cotidianas.

## RESUMO

**Objetivo:**

Mapear intervenções educativas domiciliares para cuidadores familiares de idosos pós-AVC.

**Método:**

Revisão de escopo baseada na metodologia JBI, realizada em 23 de maio de 2023. Foram utilizados o aplicativo Rayyan e Preferred Reporting Items for Systematic reviews and Meta-Analyses extension for Scoping Reviews.

**Resultados:**

Dos 1.705 estudos, foram incluídos nove publicados de 2006 a 2020: 44% das intervenções foram educativas teórico-práticas; 77,7% eram ensaios clínicos randomizados; e a intervenção “presencial” (56%) foi a mais comum, realizada por enfermeiros em 88,9% dos casos. Foram realizadas de três a 15 sessões de 42 minutos. Os conteúdos educativos foram organizados em dez categorias, divididas em educação voltada ao cuidado do idoso e autocuidado ao cuidador.

**Conclusão:**

As intervenções educativas identificadas fortalecem os conhecimentos e habilidades dos participantes em áreas como educação, cuidado, comunicação, autogestão, reabilitação e nutrição, bem como autocuidado para auxiliar com segurança os idosos em suas atividades diárias.

## INTRODUCTION

Stroke continues to be a major public health problem worldwide^(1)^ that generates highly disabling neurological lesions in the survivor. In 2022, it was estimated that 14 million people worldwide suffered a stroke, with a frequency of one every 53 seconds and a mortality of one person every 3.3 minutes^(2)^. Currently, there are more than 100 million people living with disabilities due to stroke, mostly older adults^(3)^, and 143 million years of healthy life have been lost, 40.8 million disability-adjusted life years and 4.5 million years lived with disability worldwide in people under 70 years of age^(4)^.

Stroke can cause cognitive and/or functional impairment that negatively impacts communication, swallowing and mobility, among other limitations, and as a consequence, restrictions occur in survivors’ basic and instrumental activities of daily living^(5)^. In this regard, caring for survivors after a stroke requires long-term care^(6)^, which implies the need to have a family caregiver (FC) who is responsible for meeting the needs related to physical and/or cognitive disabilities^(7)^.

The FC is that person who belongs to the family unit over 18 years of age and who is responsible for providing care, physical, emotional, social and spiritual assistance^(8,9,10)^ to older adults under disability and dependency in the hospital or home. FC make decisions and take over self-care, and must have adequate understanding of the disease and the care to be provided at home^(11)^.

Studies report that caregivers are forced to learn to care for patients through trial and error, since they are discharged without caregivers receiving adequate education. Nursing action is important to promote knowledge and development of skills to face complex situations during the transition and adaptation of care at home^(12,13)^.

Health education refers to the discipline in charge of guiding and structuring educational activities with the objective of positively impacting the knowledge, practices, habits of people and communities in relation to their health^(14)^. An educational intervention in the home environment is a strategic and planned teaching-learning process, which seeks to promote knowledge, skills and attitudes with teaching aids. Information and communication technologies are used so that individuals or families can enrich the learning process and make informed decisions about their healthcare or that of a family member^(15)^.

Adapting educational interventions to the home is of utmost importance, given that the transition from hospital to home represents a challenging period for both stroke and FC survivors, which can cause adverse events, hospital readmissions and delayed recovery in older adults as well as psychological stress, social isolation and low quality of life for both survivors and caregivers^(16)^.

Educational interventions from various approaches must be adapted to the specific needs of caregivers and the recovery phase of the stroke survivor. In the first phase, acute, it extends from hospital discharge until the first six months in the community, and the chronic phase begins six months after returning to the community^(17)^.

It is crucial to understand that proper care can prevent long-term negative consequences, such as a decrease in quality of life, mood disorders, physical and social impairments for the dyad^(18)^. Currently, there is no standard educational intervention for FC in older adults after a stroke^(19,20,21)^. Therefore, this scoping review aimed to map home-based educational interventions for FC in older adults after a stroke.

## METHOD

### Study Design

This scoping review was developed under the JBI methodology, and the search was carried out on May 23, 2023. The review protocol is registered in the Open Science Framework (OSF) at the link https://doi.org/10.17605 /OSF.IO/VH759^(22)^.

The study question formulation occurred through the acronym PCC (Population = FC of older adults; Concept = educational interventions; and Context = home)^(23)^.

### Review Question

What are the educational interventions at home for FC in older adults after a stroke?

### Inclusion y Exclusion Criteria

We included FC of older adults after a stroke, educational interventions, for assistance, care and rehabilitation, adapted and/or personalized with brief, individual, group, theoretical-practical approaches, skills development, psychoeducation and peer support, carried out at caregivers’ homes.

We excluded paid non-professional caregivers, educational interventions in hospitals or rehabilitation units as well as in community health centers, and interventions aimed at FC of patients with disabilities and dependency not related to stroke.

### Search Strategy

The search strategy followed three steps with the objective of finding both published and unpublished studies. First, an initial limited search was performed in PubMed, followed by analysis of the keywords contained in the title and abstract as well as the indexed keywords. A second search was then conducted using all keywords and index terms identified in all included databases. Finally, the search for gray literature was carried out, which involves reviewing the list of studies of interest in national repositories, combined with reviewing the references of all identified reports and articles to find additional relevant studies. It should be noted that there were no limitations regarding language and publication date in the search.

### Databases Used

The search was performed in the following databases: MEDLINE (access via PudMed); Cumulative Index to Nursing and Allied Health Literature (CINAHL); Scopus; ERIC; ScienceDirect; VHL Portal (BDENF, LILACS, IBECS); PsycInfo; Excerpta Medica Database (EMBASE). The gray literature search was carried out in the CAPES Thesis and Dissertation Catalog, NDLTD Database and Google Scholar. In this review, the descriptors and/or keywords referenced in the thesaurus of Descriptors in Health Sciences (DeCS), Medical Subject Headings (MeSH), in addition to the Elsevier Authorized Life Sciences Thesaurus (EMTREE), combined with the Boolean connectors “AND”, “OR” and “NOT”, were used, with the purpose of specifying logical relationships between search terms and improving the accuracy and relevance of search results (Chart 1).

**Chart 1:** Database search strategy – México, 2023.

Databases	Search strategy	Retrieved files
PubMed	Caregiver* AND education AND (stroke OR hemiplegia)	537
CINAHL	(MH “Hemiplegia”) OR TI (hemiplegia or hemiparesis or hemiparetic or hemiplegic OR hemiplegias) OR AB (hemiplegia or hemiparesis or hemiparetic or hemiplegic OR hemiplegias) OR (MHxxx “Stroke”) OR TI (stroke or “cerebrovascular accident” or cva or “cerebral vascular event” or cve or “transient ischaemic attack” or tia) OR AB (stroke or “cerebrovascular accident” or cva or “cerebral vascular event” or cve or “transient ischaemic attack” or tia) AND (MH “Caregivers/ED”) OR TI ((caregiver* OR carer*) AND education) OR AB ((caregiver* OR carer*) AND education) AND Restringir por SubjectAge: – all adult	228
Scopus	TITLE-ABS-KEY-AUTH (caregiver* AND education AND (stroke OR hemiplegia)) AND TITLE-ABS-KEY-AUTH (“INTERVENTION”)	302
ERIC	“family caregiver” AND education AND stroke	02
Science Direct	(Caregiver OR carer) AND “educational intervention” AND (stroke OR hemiplegia)	20
VHL (BDENF, LILACS, IBECS)	(family caregiver) AND ((education OR intervention)) AND ((stroke OR hemiplegia)) AND (db:(“BDENF” OR “LILACS” OR “IBECS”))	46
PsycInfo	**Any Field**: “family caregiver” *AND* **Any Field**: education *AND* **Any Field**: stroke	19
EMBASE	(‘family caregiver’/exp OR ‘family caregiver’) AND (‘education’/exp OR education) AND (‘cerebrovascular accident’/exp OR ‘cerebrovascular accident’) AND (home OR domiciliar OR outpatient	281
CAPES Thesis and Dissertation Catalog	(educação OR intervenção) AND “cuidadores familiares”	31
NDLTD Database	“family caregiver” AND (education OR intervention) AND (stroke OR hemiplegia) AND (elder* OR aged OR older OR senior OR geriatr*)	17
Google Scholar	“family caregiver” AND (education OR intervention) AND (stroke OR hemiplegia) AND (elder* OR aged OR older OR senior OR geriatr*) AND Filetype: doc OR RTF OR PDF OR html	191

**Source:** own elaboration, 2023.

After the search, the information was imported into the Rayyan application (Intelligent Systematic Review)^(24)^, using the Preferred Reporting Items for Systematic reviews and Meta-Analyses extension for Scoping Reviews (PRISMA-ScR) for the presentation of this manuscript^(25)^. The first step was to eliminate duplicate information according to the methodological framework used. Subsequently, the files’ title and abstract were read, applying the inclusion and exclusion criteria. For this phase, it was necessary for two reviewers to assess the files independently and, in case of any divergence, it was resolved by a third reviewer.

**Figure 1:**
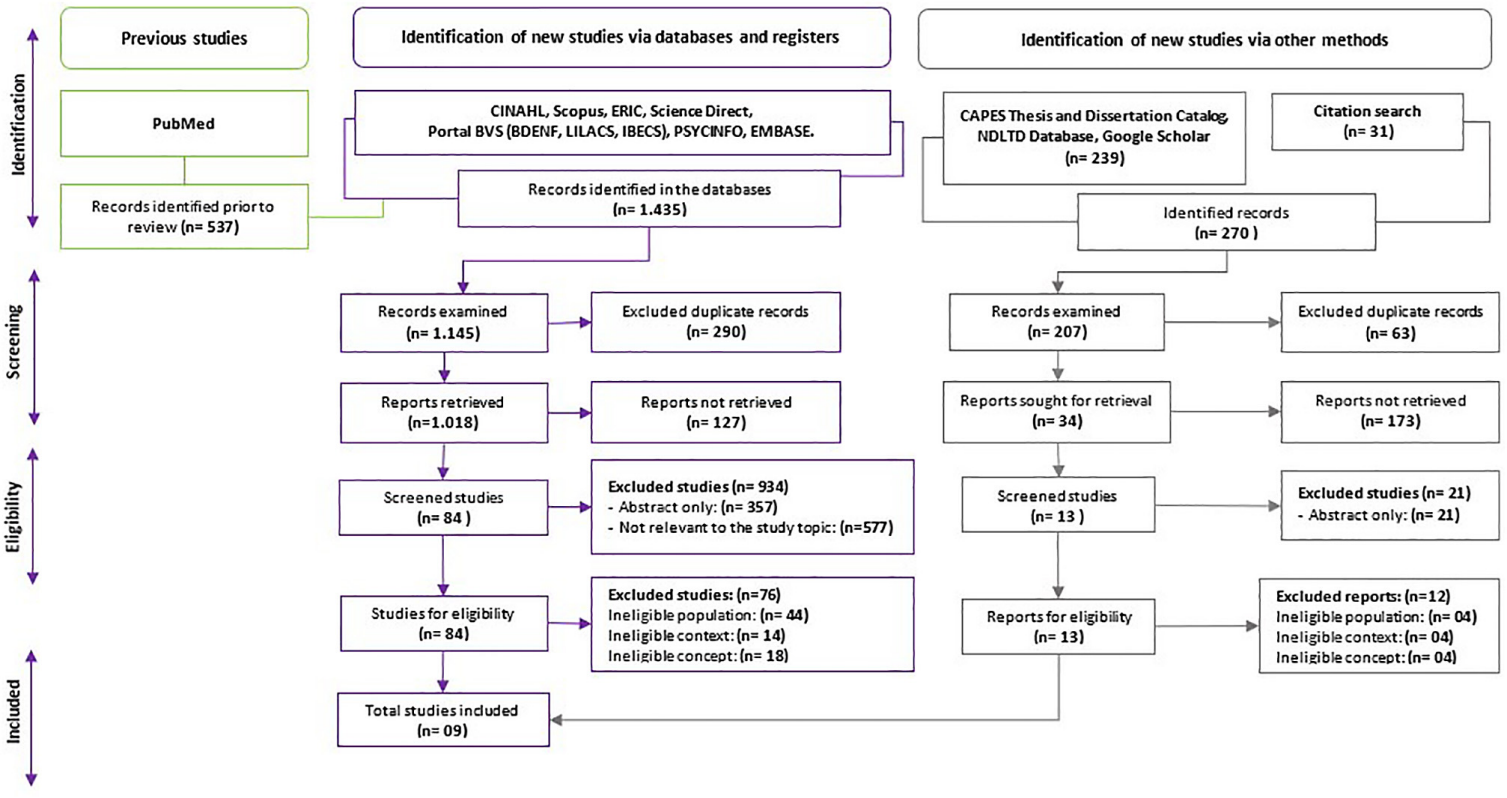
Flowchart on the steps of selection of educational interventions for family caregivers of older adults after a stroke, adaptation of the PRISMA-ScR model, 2023.

Hence, the articles were read in their entirety applying the inclusion and exclusion criteria, and simultaneously a tool designed by the authors based on the form suggested by the JBI manual was used. The numerical description of the data selection process is described in the PRISMA 2020 flowchart for updated systematic reviews that included searches in databases, registries and other sources in Figure 1.

The information that was extracted includes study identification, author/s data, year of publication, country and journal, in addition to the title, objective, methodological design, number of participants, number of sessions, dosage, empirical indicators used and follow-up time. Following the results, findings relevant to practice were highlighted. The bases of these can be found as a complement in the SciELO Data indexed to REEUSP.

### Data Analysis and Presentation

To analyze the data, descriptive statistics were used through the use of frequencies and percentages. The information collected was presented in the form of charts with detailed information for understanding.

## RESULTS

In the database search, 1,435 articles were identified, and in gray literature search, 270 documents were identified, totaling 1,705 files. After removing duplicates, 1,352 files were reviewed in which the inclusion and exclusion criteria were applied. Of these, 1,052 files were recovered; the abstract was analyzed according to readability criteria; 97 full texts were selected for a more detailed assessment, of which 88 were excluded for not addressing the topic of study, obtaining a final sample of nine documents (Figure 1).

When analyzing the information of the articles, in relation to the year of publication, it was identified that it covered between 2006 and 2020. Moreover, four studies were carried out in the United States (44%)^(26–29)^ two in Brazil (22%)^(30,31)^ and one in China, the United Kingdom and Egypt (11%)^(17,32,33)^, respectively. Likewise, 88.8% of studies were articles^(17,26–29,31–33)^ and 11.1% were a thesis^(30)^.

Of the nine studies analyzed, 44.4% had as their main objective to carry out theoretical/practical educational interventions^(26,30–32)^; 33.6% were interventions with a theoretical approach^(17,28,29)^; and 22.4% were educational interventions for guidance and support^(27,33)^.

In terms of methodological design, seven (77.7%) were Randomized Clinical Trials (RCT)^(17,27,29–33)^ and in two (22.3%) the methodological design was not identified. However, the type of intervention performed was described, and the results are presented^(26,28)^. In relation to how the intervention was carried out, face to face was the most common (56%)^(26,30–33)^, followed by the use of telephone call, website (33%)^(17,28,29)^ and asynchronous phone call/online messaging (11%)^(27)^.

It should be noted that, of the nine studies, eight (88.9%) were implemented by nurses^(17,26–31,33)^ and one (11.1%) by a multidisciplinary team made up of nurses, doctors, psychologists and physiotherapists^(32)^. In relation to teaching resources, 56% delivered the intervention manual^(26,27,30–32)^; 33% used the website^(17,28,29)^; and 11% combined the delivery of manuals plus the use of instructional videos^(33)^.

In relation to the number of sessions, it varied between three and 15. Regarding the time used per session, it fluctuated between 23 and 120 minutes, having an average of 42 minutes. On the other hand, the duration of the study ranged between four and 26 weeks (Chart 2).

**Chart 2:** Characteristics of included studies on educational interventions for family caregivers of older adults after stroke – México, 2023.

ID	Country	TEI	MD	MoD	Facilitator	ETR	No. S	Dosis	ST
Schure et al.^(26)^	United States	Theoretical/practical	N/d	In-person	Nurses	Intervention manual	8	120 m	10 W
McLennon et al.^(27)^	United States	Educational/guidance	RCT	Telephone line	Nurses	Intervention manual	9	23 m	N/d
Baltar et al.^(30)^	Brazil	Theoretical/práctica	RCT	In-person	Nurses	Intervention manual	3	40 m	4 W
Fu et al.^(32)^	China	Theoretical/practical	RCT	In-person	Nurses, psychologists, doctors	Intervention manual	9	45 m	9 W
LeLaurin et al.^(28)^	United States	Theoretical	N/d	*Website*	Nurses	*Website* for caregivers	15	N/d	N/d
Patchwood et al.^(33)^	United Kingdom	Educational/support	RCT	In-person	Nurses	Intervention manual – instructional videos	10	120 m	6 W
Elsheikh et al.^(17)^	Egypt	Theoretical	RCT	In-person/telephone line	Nurses	Online educational resource	10	120 m	26 W
Kottwitz et al.^(31)^	Brazil	Theoretical/practical	RCT	In-person	Nurses	Intervention manual	3	40 m	4 W
Quinn et al.^(29)^	United States	Theoretical	RCT	Asynchronous phone line/online messaging	Nurses	*Website* for caregivers	9	N/d	N/d

*ID = identification data (author, year of publication); TEI = type of educational intervention; MoD = mode of delivery; MD = methodological design; RCT = Randomized Controlled Trial; ETR = educational teaching resource; No. S = number of sessions; m = minutes; N/d = no data; ST= study time; W = weeks.

**Source:** own elaboration, 2023.

In total, 1,359 FC participated in the nine studies. Of this group, 232 (23.77%) were men and 1,036 (76.23%) were women, with an average age of 56.34 years. In relation to the family bond with older adults, it is observed that 70.9% were spouses, 21.7% were children, 3.5% were daughters-in-law, and 3.7% had another type of family bond.

As for FC’s marital status, 90.9% are married, 7.2% are single, and 1.5% are widowed. Concerning the educational level, it is identified that 10% do not know how to read and/or write; 16.8% have primary education; 21.6% have secondary education; 25.3% have a high school education; 25.5% have a university education; and 0.6% have completed graduate studies. Regarding occupation and/or work activity, 5% report being employers; 6.6% report being unemployed, 27.7% are workers and, for the most part, 48.8% are retired.

In relation to the educational content provided to participants during the sessions, they were organized into a total of 10 categories, divided into two groups. Categories one to seven are intended for FC, with the purpose of strengthening their knowledge, skills and strategies related to home care for older adults. The remaining categories focused on self-care and strategies for coping with challenges and organization for the FC themselves (Chart 3).

**Chart 3:** Categories of educational interventions for family caregivers of older adults after a stroke – México, 2023.

Educational categories aimed at caring for older adults	Study that integrates the component								
	Schure et al.^(26)^	McLennon et al.^(27)^	Baltar et al.^(30)^	Fu et al.^(32)^	LeLaurin et al.^(28)^	Patchwood et al.^(33)^	Elsheikh et al.^(17)^	Kottwitz et al.^(31)^	Quinn et al.^(29)^
Knowledge and education about stroke	X	X	X	X	X	X	X	X	X
Assistance and care in basic and instrumental activities of daily living	X	X	X	X	X	X	X	X	X
Effective communication	X		X	X	X		X	X	X
Self-management of health services			X	X	X	X		X	X
Physical and language rehabilitation			X		X		X	X	X
Nutrition/feeding			X	X	X			X	X
Risks prevention			X		X			X	X
**Educational categories aimed at family caregivers**									
Self-care and well-being			X	X	X		X	X	X
Strategies to face challenges	X	X	X	X	X	X		X	X
Organization of the caregiver role	X		X		X	X	X	X	X

**Source:** own elaboration, 2023.

The creation of these categories was based on an exhaustive analysis of the educational components present in each intervention. From this analysis, codes were generated that were subsequently grouped with the aim of addressing the majority of the contents related to the specific challenges of each situation. Since some interventions included a variety of components, some of them were considered complex interventions^(28–31)^.

It is relevant to highlight that 100% of interventions comprehensively addressed the educational components related to stroke, its sequels and the impact on survivors’ dependency and the impact on FC, also incorporating teaching and/or guidance on assistance and care in basic and instrumental activities of daily living, respectively.

Furthermore, 77% of interventions integrated effective communication between caregivers and older adults^(17,26–32)^. In relation to the management of health services for continuous care of stroke, physical sequels and other chronic diseases, 66% considered this element within their educational components^(28–33)^. As for the physical rehabilitation, nutrition/food category, 55% integrated it^(17,28–31)^ and 44% integrated the risk prevention at home category^(28–31)^.

In the second group of categories aimed at FC, 88% of studies integrated the strategies to face challenges during care for older adults at home category^(26–33)^, followed by 77% that integrated the caregiver role organizational component^(17,26,28–33)^. Finally, only 66% of studies included the self-care and well-being category^(17,28,30–32)^.

## DISCUSSION

Current literature shows that approximately half of the interventions are designed to promote FC’s acquisition of practical home care knowledge and skills^(26,30–32)^. On the other hand, the other half of interventions focus on improving knowledge, guidance and support through telephone lines or websites, both synchronously and asynchronously, at FC’s home^(17,27–29)^.

The educational interventions carried out mostly had RCT as a methodological design; of these, the studies^(26–28)^ reported significant differences between the groups. However, the results of studies^(17,29–33)^ did not show significant changes in the variables measured in intervention groups. One possible explanation is that it is still unclear how best to support caregivers and survivors after a stroke, given the complexity of the phenomenon and the diversity of needs and challenges at home^(33)^.

Of the previous interventions, only the studies^(17,31)^ reported significant changes in the psychological-social domains and social relationships and autonomy, respectively, which attribute the changes to the intervention carried out. It should be noted that the problem-solving study^(29)^, published in 2023, only found a significant change in activities of daily living of survivors after 11 weeks of application of the intervention. An important finding is that participants report that it is necessary to reinforce the information in follow-up sessions to strengthen and/or improve practical care skills^(27,29)^.

It is important to highlight that all interventions share the common characteristic of instructing FC in the assessment and understanding of the needs of older adults with disabilities and dependence after a stroke, with the aim of providing care based on humanized responses. This quality is essential since it is part of the essence of care. Furthermore, the majority of these interventions have been developed by nursing professionals, with more than 95% being carried out by nurses.

In relation to the educational categories oriented to FC to care for older adults at home, most studies agree that the first category should provide knowledge and education about stroke. The objective is to answer questions related to the sequels that arise in older adults after a stroke, in addition to the changes and adaptive coping strategies during the acute phase in the first six months. Thus, we sought to respond to the need for effective adaptation of older adults during the chronic phase at home in the physical, social and emotional dimensions^(17,26–33)^.

Education about stroke should not be limited to understanding the condition and the injuries it causes in the survivor. It is necessary to raise awareness in FC about the disability and dependence that older adults will have after suffering a stroke. In this way, caregivers will be prepared to provide the assistance and supervision required in survivors’ basic and instrumental activities of daily living^(26,28–30)^. Thus, the intervention aims to improve quality of life, prolong life and maintain the dignity of people living with disabilities and dependency after a stroke^(31,32)^. Therefore, contributing to the generation of skills in FC will allow it to implement techniques to assist older adults in a safe and dignified manner^(27)^, which will promote autonomy and self-esteem for older adults, and will help prevent physical injuries, pressure injuries, malnutrition and other problems^(17,33)^.

To achieve autonomy and self-esteem in older adults, several studies^(17,26–32)^ have considered it necessary for caregivers to design effective communication strategies with older adults based on the resources provided in the interventions. This is based on the premise that brain injury does not generate loss of hearing or ability in basic psychological processes, which are fundamental for older adults’ mental health and well-being, considering that effective communication and interaction allow older adults to feel understood, respected and maintain their dignity.

Furthermore, other studies^(28–33)^ have considered it crucial that FC learn to manage the health services that their family members need on their own. Additionally, physical, language, caregiving and nutrition rehabilitation can empower caregivers, teaching them to manage and learn on their own, with the goal of improving health and well-being outcomes for older adults in their care.

Caregivers informed about existing resources and how to request them can ensure that their family members receive timely and appropriate care. Likewise, this information allows them to request education for the prevention of health risks for older adults while they remain at home. Hence, four studies integrated the educational component as an important resource for caregivers with the aim of reducing hospital readmissions, in addition to self-management that reduces caregiver stress^(28–31)^.

When they do not know how to access certain services, caregivers can feel frustration and helplessness^(29,31)^. Providing the tools to proactively ask for help gives caregivers a sense of control and self-efficacy which can prevent caregiver burnout^(28–31)^.

Although these interventions were created and applied in diverse geographical contexts around the world, they integrated into their educational interventions various categories aimed at FC, because stroke survivors’ needs remain similar due to the nature of the condition and its sequels.

However, FC’s needs present considerable variability, since the factors studied focus on overload^(17,26,30)^, quality of life for both caregivers and survivors^(17,26,31,32)^, care and coping with caregivers’ comprehensive health in relation to tension, anxiety and depression^(33)^. In this regard, the interventions are also focused on problem resolution^(29)^ and the empowerment of caregivers in care^(27,28)^.

Self-care and well-being interventions, derived from the fact that the role of caregiver can be very physically and emotionally demanding^(17,28–31)^, included the category of teaching appropriate care techniques with stress reduction and injury prevention in the caregivers themselves.

Thus, the studies used strategies to face challenges for caregivers during home care for older adults with disabilities and post-stroke dependency derived from the multiple physical and emotional challenges for caregivers^(26–33)^.

Coping strategies allow to better manage stress, overload, isolation and changes in family dynamics^(28)^. Additionally, it benefits the development of skills to provide the required care and promote their own well-being^(32)^, incorporating these strategies in caregiver education, increasing their resources and capabilities to take over the role^(28)^ and reducing the risk of negative outcomes for both caregivers and care recipients^(33)^.

The incorporation of the organization of the caregiver role in educational interventions category complements FC preparation, since it allows them to readjust the home to dependent persons’ needs, establish care routines, plan and delegate tasks, manage time and seek support networks^(17,26,28–33)^. Hence, caregivers can better organize their new role, which is key to preventing overload, promoting their well-being and providing quality care to older adults^(28)^. Furthermore, it enhances caregivers’ skills to function at home.

As such, FC recognize their work as not only relevant for dependent older adults, but also for itself. This allows to transcend the vision of care and find a deeper meaning that goes beyond everyday work and difficulties^(17)^.

Transcendence implies that caregivers can find satisfaction, understanding, and patience through the care process. This strengthens the bond between older adults and FC, and connects them in the spiritual dimension of care, beyond the physical. Seen in these terms, the limited view of caregiver role as a burden or responsibility^(31)^ is overcome to assume it as an opportunity for personal growth, generating a significant bond that adds value to the lives of both^(17)^.

Among the limitations of this study, it was identified that professional or paid caregivers’ specific needs were not addressed. Furthermore, due to the diversity of interventions carried out in different study methods, it was not possible to assess the methodological quality of studies due to the methodology itself.

The physical and cognitive consequences left by stroke in survivors require the design of interventions according to caregivers’ felt needs at home, in order to generate greater positive effects in the transition process and adoption of their new role. More research is needed to determine optimal approaches to prepare and support FC during the transition from hospital to home.

## CONCLUSION

Home-based educational interventions aimed at FC of older adults with stroke are limited. Those identified in this study sought to strengthen participants’ knowledge and skills in areas such as education, assistance and care of older adults, effective communication, self-management, rehabilitation, nutrition and risk prevention. On the other hand, the interventions focused on caregivers were aimed at promoting their self-care and well-being, providing strategies to face challenges and organize their role, in order to prepare them to safely assist older adults in their activities of daily living.

The results showed that the interventions mainly focus on strengthening caregivers’ knowledge and practical skills to provide quality care at home, improving their guidance and support through means such as telephone lines and websites.

A consistent finding is the importance of reinforcing the education provided through follow-up. It is also key to promote understanding of the particular needs of each survivor to provide humanized, quality care. Thus, it is necessary to investigate FC’s felt needs after stroke of survivors and design multicomponent educational interventions that allow caregivers to become aware of the importance of their role and facilitate a healthy transition towards the adoption of the role.
